# On-Line Detection Method of Salted Egg Yolks with Impurities Based on Improved YOLOv7 Combined with DeepSORT

**DOI:** 10.3390/foods13162562

**Published:** 2024-08-16

**Authors:** Dongjun Gong, Shida Zhao, Shucai Wang, Yuehui Li, Yong Ye, Lianfei Huo, Zongchun Bai

**Affiliations:** 1 College of Engineering, Huazhong Agricultural University, Wuhan 430070, China; 2 Wuhan Vocational College of Software and Engineering (Wuhan Open University), Wuhan 430205, China; 3 Institute of Facilities and Equipment in Agriculture, Jiangsu Academy of Agricultural Sciences, Nanjing 210014, China; 4 Key Laboratory of Protected Agriculture Engineering in the Middle and Lower Reaches of Yangtze River, Ministry of Agriculture and Rural Affairs, Nanjing 210014, China

**Keywords:** salted duck egg yolk, online processing, impurities, attention mechanism, object detection, tracking

## Abstract

Salted duck egg yolk, a key ingredient in various specialty foods in China, frequently contains broken eggshell fragments embedded in the yolk due to high-speed shell-breaking processes, which pose significant food safety risks. This paper presents an online detection method, YOLOv7-SEY-DeepSORT (salted egg yolk, SEY), designed to integrate an enhanced YOLOv7 with DeepSORT for real-time and accurate identification of salted egg yolks with impurities on production lines. The proposed method utilizes YOLOv7 as the core network, incorporating multiple Coordinate Attention (CA) modules in its Neck section to enhance the extraction of subtle eggshell impurities. To address the impact of imbalanced sample proportions on detection accuracy, the Focal-EIoU loss function is employed, adaptively adjusting bounding box loss values to ensure precise localization of yolks with impurities in images. The backbone network is replaced with the lightweight MobileOne neural network to reduce model parameters and improve real-time detection performance. DeepSORT is used for matching and tracking yolk targets across frames, accommodating rotational variations. Experimental results demonstrate that YOLOv7-SEY-DeepSORT achieves a mean average precision (mAP) of 0.931, reflecting a 0.53% improvement over the original YOLOv7. The method also shows enhanced tracking performance, with Multiple Object Tracking Accuracy (MOTA) and Multiple Object Tracking Precision (MOTP) scores of 87.9% and 73.8%, respectively, representing increases of 17.0% and 9.8% over SORT and 2.9% and 4.7% over Tracktor. Overall, the proposed method balances high detection accuracy with real-time performance, surpassing other mainstream object detection methods in comprehensive performance. Thus, it provides a robust solution for the rapid and accurate detection of defective salted egg yolks and offers a technical foundation and reference for future research on the automated and safe processing of egg products.

## 1. Introduction

China is a leading producer and consumer of poultry eggs. In recent years, production has steadily increased to meet growing market demand [1]. In 2022, China’s poultry egg production reached 34.56 million tons, representing a 1.38% year-to-year increase and accounting for 36.30% of global output [2]. Among the various products derived from poultry eggs, salted egg yolk is of significant importance in China’s poultry egg industry. Renowned for its distinctive taste and nutritional value, salted egg yolk is a key ingredient in numerous specialty food products [3]. The rapid advancement of pre-prepared food technology has further amplified the demand for salted egg yolks [4]. Consequently, there is a need for the development of efficient and safe processing technologies for salted egg yolks. Typically produced from duck eggs through specific curing processes, salted egg yolks may contain impurities due to variations in egg quality and size during shelling. Current high-speed egg-breaking devices often embed small eggshell fragments into the salted egg yolks, posing safety risks in subsequent food processing. Therefore, efficient and precise detection of salted egg yolks with impurities has become crucial for advancing salted egg food processing technologies.

Traditionally, the detection and removal of salted egg yolks with impurities have relied heavily on manual labor, which is characterized by high labor intensity, low efficiency, and insufficient precision. This approach is inadequate for meeting the increasing market demands. With the diversification of non-destructive testing technologies, researchers have explored various methods for quality inspection and food processing, including computer vision, hyper-spectral/near-infrared imaging, physicochemical analysis, and mechanical principles, leveraging multiple physiological characteristics of salted egg yolks.

Chen et al. [5] addressed the challenge of detecting fluid-state salted duck egg yolks by introducing a location-aware circular convolution and vision transformer to enhance ConvNext-T, proposing a non-destructive quality detection method with an accuracy of 96.83%. Xu et al. [6] utilized a hyperspectral imaging system to gather spectral data from sliced salted duck eggs and developed prediction methods for moisture, lipid, and salt content using partial least squares regression, enabling accurate measurement of various indicators across different curing periods. Chen et al. [7] proposed a model for detecting salt content in both salted duck egg yolks and egg whites by employing three spectral transformations (Savitzky–Golay, continuum removal, and first-order derivation) and optimal feature wavelength extraction based on sensitive spectral factors. Tian et al. [8] established a non-destructive rapid detection model for key quality indicators of salted duck eggs (yolk moisture content, yolk sodium chloride concentration, and salted egg yolk index) based on near-infrared spectroscopy. Long et al. [9] investigated the impact of varying curing conditions on lipid forms and oxidation rates in egg yolks, revealing that increased curing temperature and extended curing time significantly enhance the yolk index, lipid content, and saponification value of egg yolks. Additionally, the amount of salt used in curing significantly promotes the saponification value of lipids in egg yolks. Li et al. [10] addressed issues related to temperature fluctuations and long curing cycles in existing equipment by designing a rapid temperature control system and a reinforced concrete structure for rapid curing of salted duck eggs. While these advancements have improved multi-quality detection and processing of salted duck eggs, research on online recognition methods for salted egg yolks with impurities remains a critical area requiring innovative solutions. Recent developments in visual perception technology, with demonstrated capabilities in egg fertilization [11,12], crack detection [13,14], gender identification [15,16], and hatch and development [17,18], suggest its potential for the rapid and accurate identification of salted egg yolks with impurities.

This study addressed the practical challenges in salted egg yolk processing by focusing on salted egg yolks with impurities. The experiment was conducted in three main stages using computer vision technology: (1) development of an salted egg yolk with impurities object detection model, YOLOv7-SEY, based on an improved YOLOv7 framework, with performance compared to other mainstream methods; (2) replacement of the YOLOv7 feature extraction network with the lightweight MobileOne, incorporating multiple CA mechanisms in the Neck section, and employing the Focal-EIoU loss function to enhance model detection accuracy; and (3) development of an online real-time detection method for salted egg yolks with impurities based on DeepSORT, with comparative analysis against other methods. The proposed online detection methods, based on improved YOLOv7 and DeepSORT, not only support advancements in salted duck egg processing technology but also offer valuable insights for the automated and safe processing of other poultry egg products.

## 2. Materials and Methods

### 2.1. Test Materials and Image Acquisition

**Figure 1:**
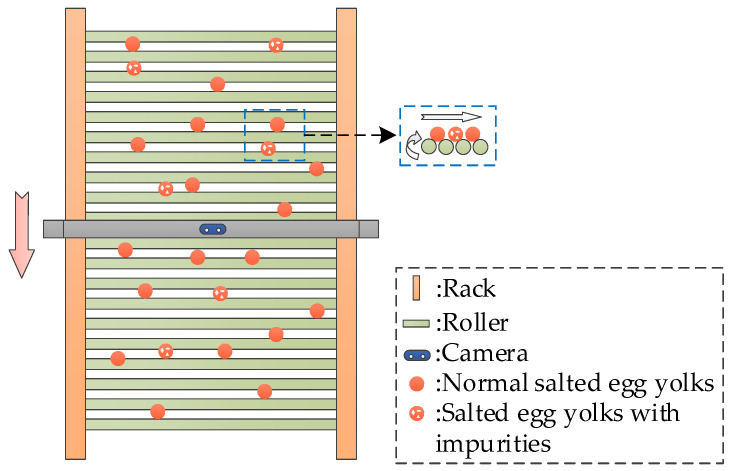
Overhead view of salted egg yolks image capture process.

**Figure 2:**
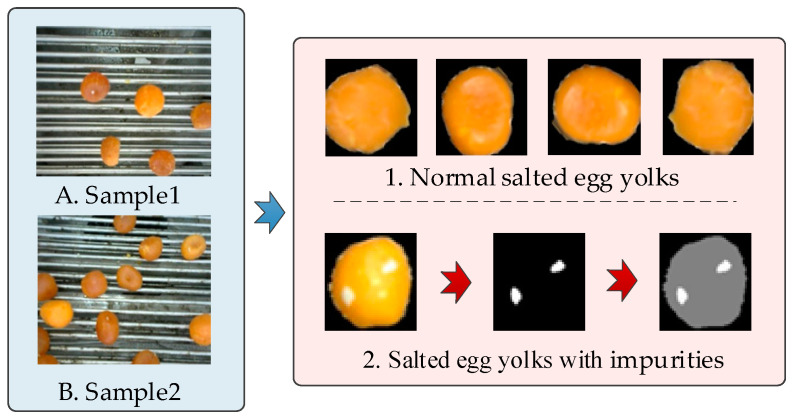
Examples of salted egg yolk images.

Uncured salted duck egg yolks were selected as test materials for this study. Sample images were collected from the production line at Hubei Shendan Healthy Food Co., Ltd. (Wuhan, China). The image capture station was positioned after a high-speed egg-breaking machine, where images of salted egg yolks on the rolling conveyor were recorded using a RealSense D435i depth camera (Intel, Santa Clara, CA, USA). The conveyor operated at a speed of 0.02 m/s, with data resolution set at 1080 × 720 pixels and a frame rate of 30 frames per second (FPS). No background boards or specific lighting sources were used to simulate real production conditions, and the camera height was adjusted to cover the central area of the conveyor belt. A total of 10,571 images of salted duck egg yolks were manually captured at fixed intervals. The image acquisition process and sample images are illustrated in Figure 1 and Figure 2.

As shown in Figure 1 and Figure 2, normal salted egg yolks and those with impurities were distributed on the conveyor belt in distinct density patterns. There was no noticeable stacking or obscuring. Salted egg yolks with impurities characterized by visible white eggshell residues displayed clear differences in color and texture compared to normal salted egg yolks. These differences provide a basis for the precise and rapid identification of salted egg yolks with impurities.

### 2.2. Preprocessing of Salted Egg Yolk Images with Impurities and Dataset Construction

**Figure 3:**
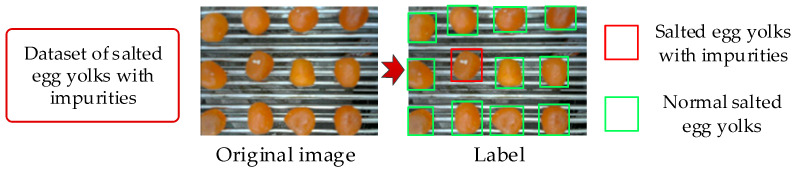
Annotation process of a dataset of salted egg yolks with impurities.

This study focused on a binary classification problem, distinguishing between salted egg yolks with and without white eggshell residues on their surfaces. Owing to the high oil content in salted egg yolks, uneven surfaces can reflect light, creating white spots that may interfere with accurate identification. To differentiate between these white spots and small white eggshell residues, experienced production staff assessed the presence of impurities from video frames, which were standardized to 640 × 640 pixels. The SAM image annotation tool was employed to construct a detection dataset for salted egg yolks with impurities, formatted according to COCO dataset standards. Normal salted egg yolks and those with impurities were labeled as “egg yolk product” and “defective product,” respectively. The dataset maintained an approximate 7:3 ratio between these two categories. The dataset composition and construction process are depicted in Figure 3 and Table 1.

### 2.3. Detection Method for Salted Egg Yolks with Impurities

YOLOv7, introduced by Wang et al. in 2022 as an advancement over YOLOv5 [19], is a single-stage object detection algorithm that directly predicts object regions, positions, and categories through regression. It offers advantages such as real-time processing, high precision, and robust generalization, making it suitable for tasks requiring efficient and accurate detection [20,21]. Given the high efficiency of the salted egg yolk production line and the uneven density distribution with localized agglomerations, this study incorporates YOLOv7-based detection for salted egg yolks with impurities, designated as YOLOv7-SEY. This method includes targeted structural enhancements to address the specific challenges of detecting impurities in salted egg yolks.

#### 2.3.1. Network Architecture for Detecting Salted Egg Yolks with Impurities

**Figure 4:**
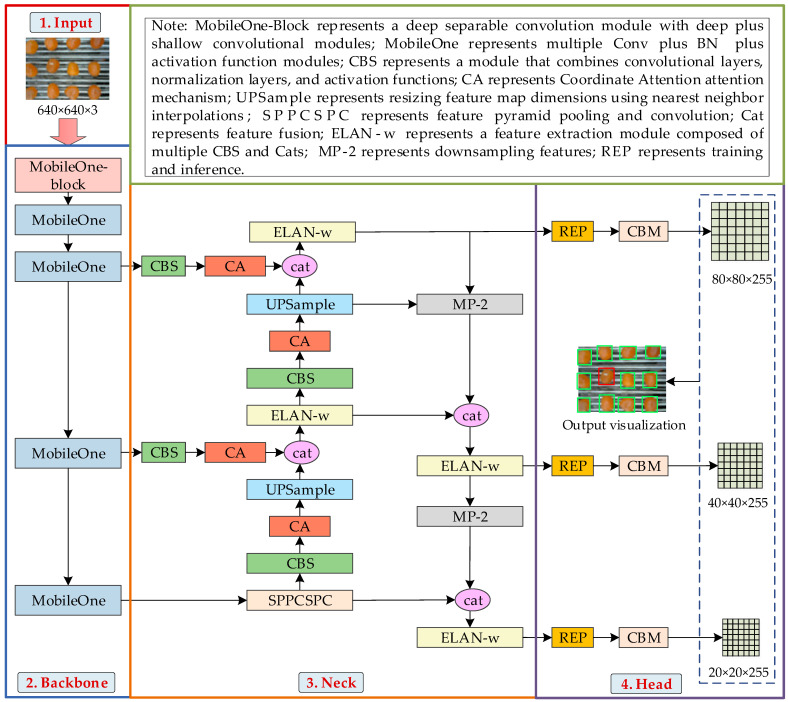
YOLOv7-SEY network architecture.

The YOLOv7-SEY network architecture builds upon the original YOLOv7 framework and comprises the Input, Backbone, Neck, and Head components. Key modifications include: (1) Replacing the original CSPDarknet53 backbone with the lightweight MobileOne [22] neural network to enhance real-time detection capabilities for salted egg yolks with impurities while preserving the detection accuracy required for online production. (2) Integrating a CA mechanism [23] after each CBS module in the Neck. Since the primary distinction between salted egg yolks with impurities and normal salted egg yolks is the presence of small white eggshell residues, the CA mechanism improves the network’s ability to extract and integrate features related to these impurities. This allows the detection model to focus on fine-grained eggshell features and differentiate between glare spots and subtle impurities, thereby enhancing detection accuracy. (3) Replacing the cross-entropy loss function with the Focal-EIoU classification loss function [24]. This modification balances the contributions of high-quality and low-quality samples to the loss, thereby improving both convergence speed and detection accuracy. The YOLOv7-SEY architecture is illustrated in Figure 4.

The Input section preprocesses salted egg yolk images with a resolution of 640 × 640 × 3 using Mosaic data augmentation, adaptive anchor box calculation, and adaptive grayscale filling before these images are passed to the Backbone. The Backbone, consisting of MobileOne-Blocks and MobileOne, performs progressive feature extraction across different scale feature maps. These maps are then adjusted and input into the feature fusion network. The Neck employs a PAFPN structure, incorporating CBS, MP-2, CA, and Spatial Pyramid Pooling with Channel Spatial Pyramid Compression (SPPCSPC). CBS extracts features, MP-2 merges features from various scales, CA enhances the network’s focus on critical features of salted egg yolks with impurities, and SPPCSPC broadens the receptive field for multi-scale feature integration. The enriched feature information is subsequently processed by the prediction network, which uses reparameterizable convolution modules to adjust channels and perform upsampling fusion across three feature layers. Instead of CIoU, Focal-EIoU is used for category prediction and bounding box anchoring specific to salted egg yolks with impurities. The final output consists of feature maps with dimensions of 20 × 20 × 255, 40 × 40 × 255, and 80 × 80 × 255, facilitating the recognition and localization of salted egg yolks with impurities.

#### 2.3.2. Lightweight Neural Network—MobileOne

**Figure 5:**
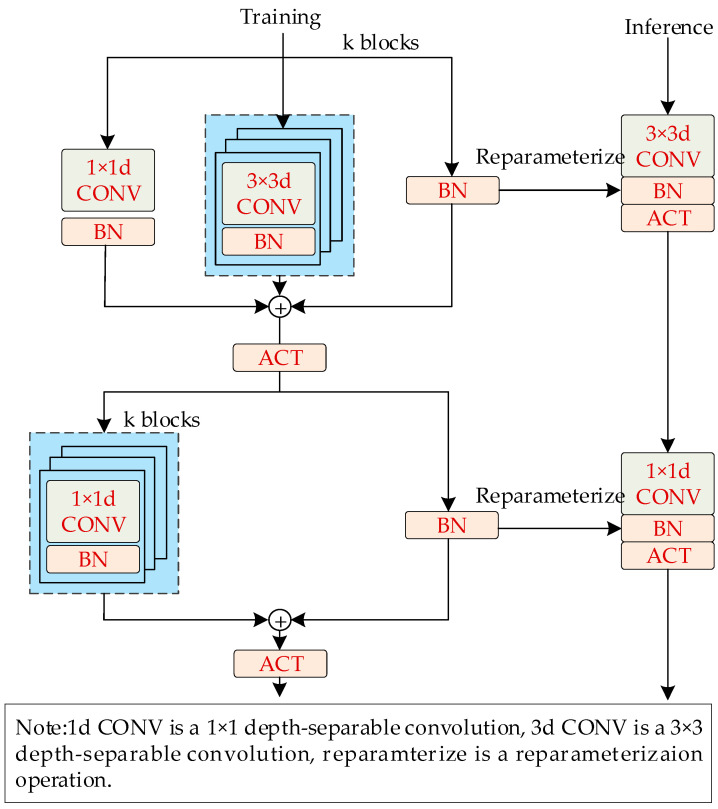
MobileOne-Block network architecture.

To address the high production efficiency demands and the need for model integration into industrial control systems or mobile devices, this study introduces the reparameterized MobileOne backbone network to streamline the detection model for salted egg yolks with impurities. MobileOne consists of multiple MobileOne-Blocks, utilizing Depthwise Convolution and Pointwise Convolution based on RepVGG reparameterization principles. The Depthwise Convolution includes 1 × 1 and 3 × 3 depth convolutions with Batch Normalization (BN) layers, organized into three branches, while the Pointwise Convolution includes 1 × 1 point convolutions with BN layers, organized into two branches. These convolutions are fused into a 3 × 3 depth convolution and a 1 × 1 point convolution through reparameterization. The structure of the MobileOne-Block network is depicted in Figure 5.

As illustrated in Figure 5, during training, the MobileOne-Block extracts extensive feature information from salted egg yolk images. In the inference phase, the multi-branch structure is reparameterized into a single-branch configuration, reducing the model parameter count and improving inference speed.

#### 2.3.3. Coordinate Attention Mechanism

**Figure 6:**
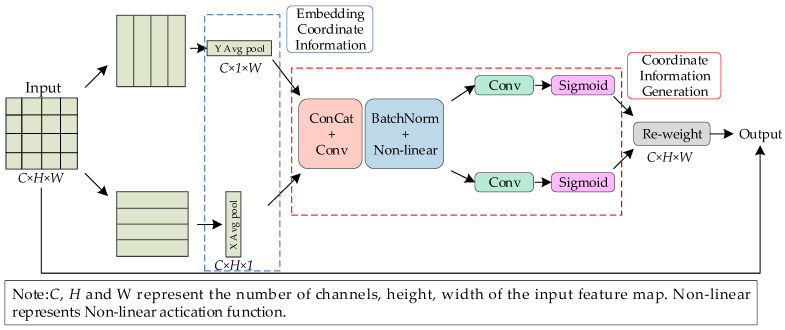
Structure of coordinate attention mechanism.

The primary distinction between normal salted egg yolks and those containing impurities is the presence of small white eggshell residues on the yolk surface. To enhance detection accuracy, the target detection network must exhibit high sensitivity to these small white eggshell features. Research indicates that incorporating attention mechanisms tailored to the characteristics of detection targets into feature extraction networks can improve the representation of primary features [25,26]. This study employed multiple CA modules within the detection network to leverage the CA mechanism for extracting, preserving, and utilizing positional information of targets. The goal was to increase the network’s focus on small white eggshells and enhance the differentiation between eggshell residues and other spots. The CA mechanism encodes and fuses spatial dependencies from both horizontal and vertical dimensions of the salted egg yolk. The structure of the CA mechanism is illustrated in Figure 6.

As depicted in Figure 6, the input feature map of size *C* × *H* × *W* was subjected to global average pooling along both height and width dimensions to generate feature maps for horizontal and vertical directions, incorporating coordinate information. This was followed by concatenation, convolution, batch normalization, and non-linear activation. Convolution and sigmoid operations were then applied separately to the height and width channels to compute directional weights, which were used to generate the coordinate information. Finally, these weights were applied to the original feature map to produce an attention-calculated weighted feature map. The input feature map of dimensions *C* × *H* × *W* was aggregated along the horizontal and vertical coordinates via global average pooling. The outputs for channel c at height h and width w can be represented using Equations (1) and (2), respectively.

(1) $$z_{c}^{h}(h)=\frac{1}{W}\sum_{0\leq i\leq W}x_{c}(h,i)$$

(2) $$z_{c}^{w}(w)=\frac{1}{H}\sum_{0\leq j\leq W}x_{c}(j,w)$$

where *W* and *H* represent the operations performed along the horizontal and vertical dimensions, respectively.

#### 2.3.4. Focal-EIoU Loss Function

This study addressed the detection of salted duck egg yolks with impurities. Compared to normal egg yolks, salted egg yolks with impurities are fewer in quantity, resulting in significant class imbalance and a long-tail distribution. Given that salted egg yolks with impurities were the detection target, the effectiveness of feature learning and bounding box localization directly affected model accuracy. Therefore, the Focal-EIoU loss was introduced to replace the CIoU loss. This loss function accounts for the overlap area between target boxes and the distance between their centers, as well as the differences in width and height of detection boxes, rather than focusing solely on the aspect ratio. This approach mitigates the class imbalance present in the dataset and balances the contribution of samples with varying impurity levels to the loss function, thereby accelerating model convergence. The computation of the Focal-EIoU loss $L_{EIoU}$ is shown in Equation (3).

(3) $$L_{EIoU}=1-IoU+\frac{\rho^{2}(b^{p},b^{gt})}{c^{2}}+\frac{\rho^{2}(w^{p},w^{gt})}{c_{w}^{2}}+\frac{\rho^{2}(h^{p},h^{gt})}{c_{h}^{2}}$$

where IoU denotes the intersection over union between the ground truth bounding box and the predicted bounding box; $\rho^{2}(b^{p},b^{gt})$ represents the Euclidean distance between the centers of the predicted and ground truth bounding boxes; $w^{p}$ and $h^{p}$ denote the width and height of the predicted box; and $w^{p},\;h^{gt}$ denote the width and height of the ground truth box. Additionally, c, $c_{w}$, and $c_{h}$ represent the minimum diagonal distance, width, and height of the bounding box, respectively.

### 2.4. Object Tracking Method of Salted Egg Yolk with Impurities Based on DeepSORT

Building upon the YOLOv7-SEY framework for detecting salted egg yolks with impurities, this study integrated DeepSORT [27] for multi-object tracking. DeepSORT, an enhancement of the SORT algorithm [28], incorporates cascade matching and state confirmation, utilizing both Mahalanobis and cosine distance metrics. This integration significantly improves tracking performance, particularly under occlusion [29,30].

**Figure 7:**
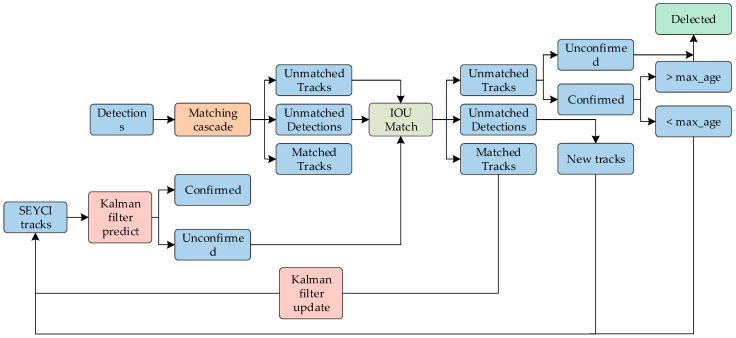
Flowchart of salted egg yolks with impurities object tracking based on DeepSORT.

DeepSORT consists of three main components: the Kalman filter for predicting and updating target states, the Hungarian algorithm for detecting and predicting IoU matches, and Mahalanobis distance for evaluating the loss index of feature embeddings. In this approach, the detection results of salted egg yolks with impurities serve as input for the DeepSORT algorithm. The Kalman filter predicts the position of the target box in the subsequent video frame, and the Hungarian algorithm matches the new predicted target box from Kalman filtering with the detected target box. This process ensures the tracking of each salted egg yolk with impurities, assigning a unique ID to each. The object tracking process based on DeepSORT is illustrated in Figure 7.

### 2.5. Evaluation Criteria

The mAP was employed as the evaluation metric to quantitatively assess the performance of the YOLOv7-SEY-based detection model, as described by Equation (4).

(4) $$\mathrm{mAP}=\frac{1}{N}\sum_{i=1}^{N}AP_{i}$$

where N represents the total number of target categories, and $AP_{i}$ is the average precision of the i-th category. The range of values for mAP is [0, 1], where a higher value indicates a better detection performance of the model. Additionally, in this paper, an IoU threshold of 0.5 is set.

For tracking performance, the effectiveness of the DeepSORT-based tracking method was evaluated using TrackEval. MOTA was used to measure the method’s ability to accurately track targets in each frame, while MOTP assessed the alignment between tracked boxes and actual boxes in each frame. These metrics are defined by Equations (5) and (6).

(5) $$\mathrm{MOTA}=1-\frac{\sum_{t}F_{N_{t}}+F_{p_{t}}+I_{DSW_{t}}}{\sum_{t}G_{T_{t}}}$$

where $G_{T_{t}}$ represents the total number of target appearances; $F_{N_{t}}$ denotes the number of target tracking failures; $F_{p_{t}}$ indicates the number of successful target trackings; $I_{DSW_{t}}$ stands for the number of times target IDs change; and t refers to the sequence of test video frames.

(6) $$\mathrm{MOTP}=\frac{\sum_{t,i}\delta_{t,i}}{\sum_{t}c_{t}}\times 100\%$$

where $\delta_{t,i}$ represents the overlap ratio between the tracking box and the detection box, while $c_{t}$ denotes the number of successfully matched targets in the current frame.

### 2.6. Experimental Environment

The experiment was conducted on a Dell tower workstation (Windows 10 Professional), utilizing the Python 3.7.1 programming language and the PyTorch deep learning framework for training and evaluating the model for detecting impurities in salted egg yolk and tracking methods. The workstation is equipped with an Intel i9-12900 CPU operating at 3.20 GHz, 128 GB of RAM, and an NVIDIA RTX 4090 GPU with 24 GB of VRAM.

### 2.7. Experimental Steps

The experiments on salted egg yolk detection and tracking, based on an improved YOLOv7 integrated with DeepSORT, involved the following steps:

(1) Images of “normal” and “with impurities” salted egg yolks were collected from actual processing scenes. Frames were extracted and processed to generate a dataset of salted egg yolks with impurities.
(2) The lightweight MobileOne backbone network replaced the original YOLOv7 backbone to reduce network parameters while maintaining accuracy and speed. Additionally, the CA module was incorporated into the Neck section, and the Focal-EIoU loss function was introduced to enhance detection accuracy. Consequently, the proposed detection network YOLOv7-SEY was developed.
(3) The YOLOv7-SEY model was trained and evaluated for improvements in accuracy and speed over the original YOLOv7. Its performance was also compared with other mainstream object detection algorithms.
(4) Building on the detection capabilities, DeepSORT was implemented for tracking salted egg yolks with impurities. The performance of this tracking method was evaluated for effectiveness.
(5) Experimental results were analyzed.

**Figure 8:**
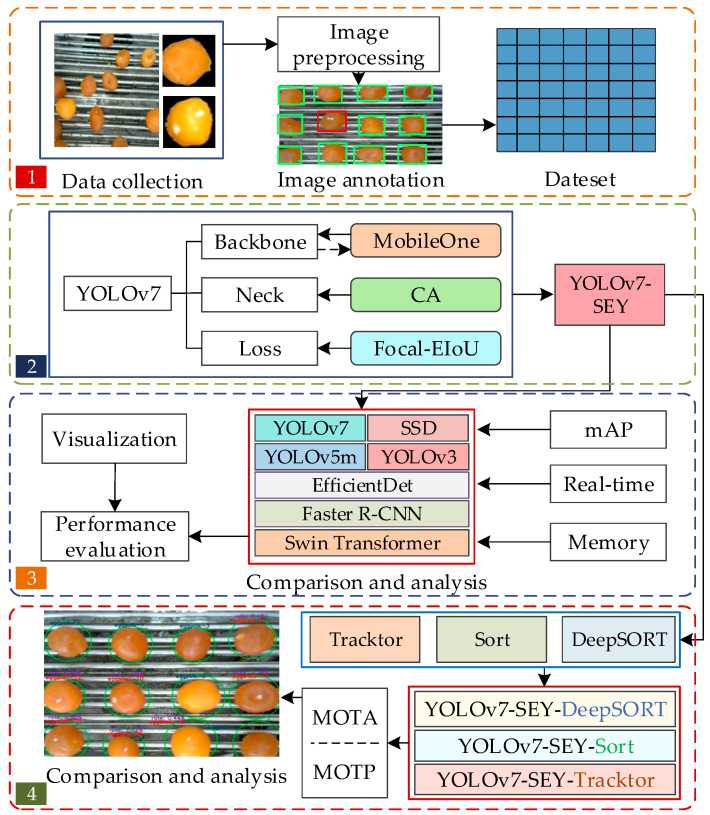
Experimental process of online detection for salted egg yolks with impurities based on improved YOLOv7 combined with DeepSORT.

The experimental procedure is illustrated in Figure 8.

## 3. Results

### 3.1. Salted Egg Yolk with Impurities Object Detection Based on YOLOv7-SEY

#### 3.1.1. Acquisition of the Salted Egg Yolk Detection Model with Impurities

Training was conducted separately on datasets containing salted egg yolks with impurities using both the original YOLOv7 and the improved YOLOv7-SEY network to derive the optimal detection model. The effectiveness of integrating the lightweight MobileOne neural network, the CA mechanism, and the Focal-EIoU loss function in enhancing detection accuracy was evaluated through a comparison of mAP values. To address overfitting and convergence issues, pre-trained weights specific to the COCO dataset were utilized, and hyperparameters were configured as detailed in Table 2.

**Figure 9:**
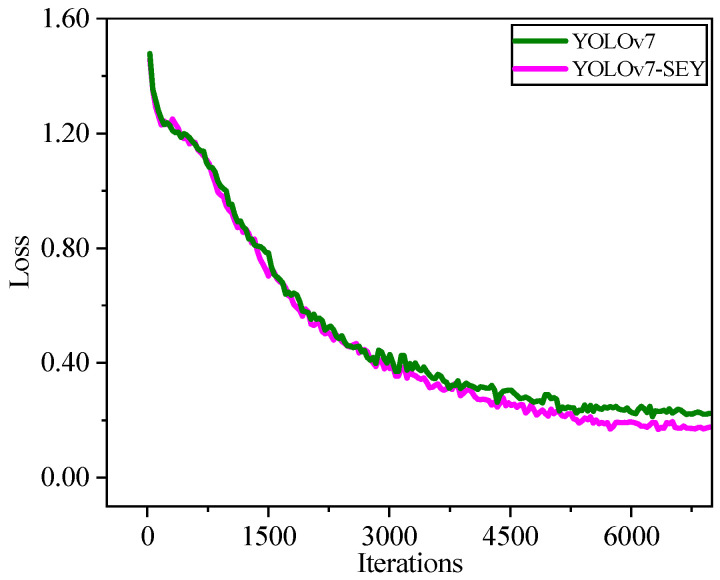
Trend in loss values with iterations during training of YOLOv7 and YOLOv7-SEY.

**Figure 10:**
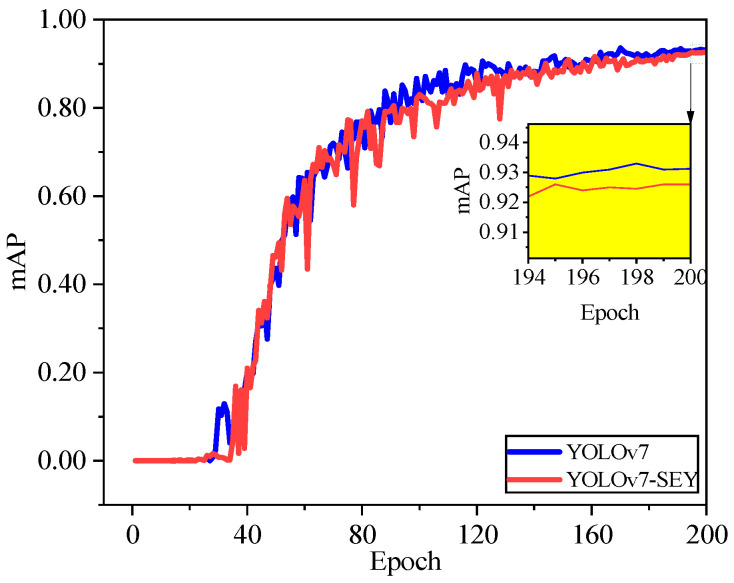
Trend in mAP values with epochs during training of YOLOv7 and YOLOv7-SEY.

As shown in Figure 9, for the dataset containing salted egg yolks with impurities, both YOLOv7 and YOLOv7-SEY exhibit a similar pattern, i.e., loss values decrease rapidly in the early stages of training and then gradually stabilize. Notably, around 3200 iterations, YOLOv7-SEY consistently exhibits lower loss values compared to YOLOv7, although both models eventually converge with minimal differences. Figure 10 illustrates that the trend in mAP values for YOLOv7 and YOLOv7-SEY mirrors the pattern observed in loss values. Both models experience rapid changes in mAP during the initial training phases, followed by stabilization and relative consistency towards the end. From Epoch 90 onwards, YOLOv7-SEY maintains a higher mAP value compared to YOLOv7. Finally, the mAP values converge to 0.926 for YOLOv7 and 0.931 for YOLOv7-SEY. These results indicate that YOLOv7-SEY demonstrates superior sensitivity to the fine shells of salted egg yolks and enhanced learning and inference capabilities for detecting impurities. YOLOv7-SEY achieves a 0.53% improvement in mAP over YOLOv7, confirming that modifications to the backbone network, the introduction of attention mechanisms, and the use of the Focal-EIoU loss function contribute to improved detection accuracy. Consequently, YOLOv7-SEY is selected for further comparative experiments on salted egg yolk detection performance.

#### 3.1.2. Detection Results and Analysis of Salted Egg Yolks with Impurities

**Figure 11:**
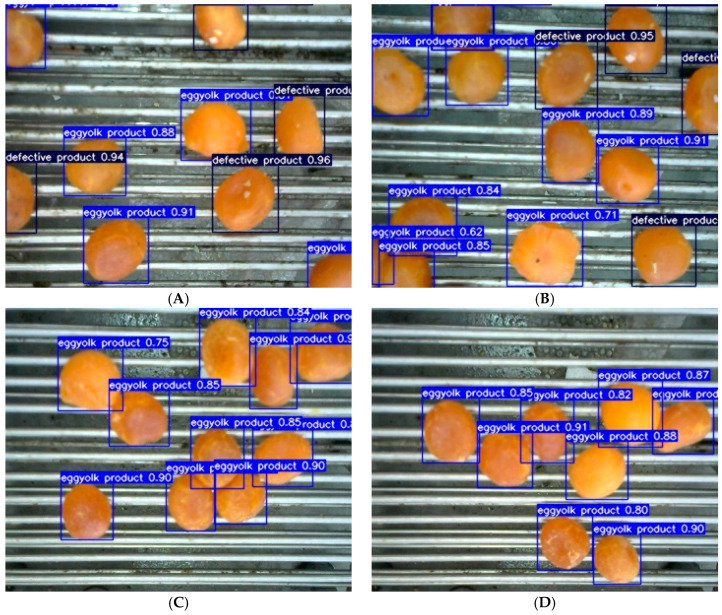
Partial detection results based on the YOLOv7-SEY model for salted egg yolks with impurities. (**A**) Sample 1. (**B**) Sample 2. (**C**) Sample 3. (**D**) Sample 4.

The YOLOv7-SEY model was employed to detect salted egg yolks with impurities in the validation set, and its performance was analyzed based on visualization results. Detection results for selected salted egg yolk images are presented in Figure 11.

Figure 11 indicates significant variations in the distribution density of salted egg yolks among the four classes (A–D) of image samples. Specifically, samples B and C exhibited higher density compared to samples A and D, with samples C and D consisting exclusively of normal salted egg yolks without impurities. The proposed model demonstrated high confidence in accurately distinguishing between salted egg yolks with impurities and normal salted egg yolks. When salted egg yolks were adhered together, the model effectively delineated the boundaries of detection boxes without omissions or false detections. Thus, the model successfully addresses challenges such as density variations, uneven class distribution, and sticky sample conditions, demonstrating robust detection capabilities.

#### 3.1.3. Generalization Ability Test

**Figure 12:**
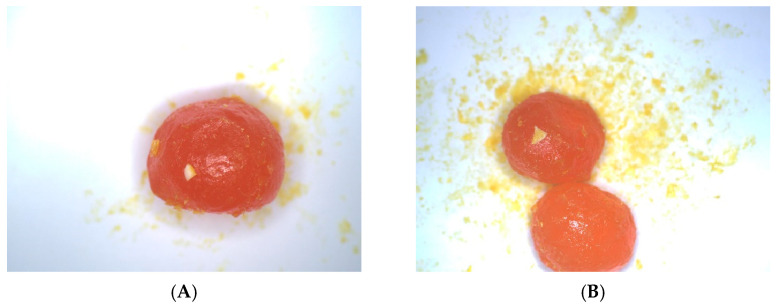
Sample examples of the generalization test dataset. (**A**) Sample 1. (**B**) Sample 2.

The effectiveness of detecting salted egg yolks with impurities across varying scenarios necessitates robust model generalization. To assess this, a generalization test dataset was created by introducing varying quantities of salted egg yolks, adjusting image brightness, and selecting images from salted egg production lines randomly. This dataset comprises 200 images with different quantities of salted egg yolks. The generalization ability of the optimal YOLOv7-SEY model for detecting salted egg yolks with impurities was then evaluated using this dataset. An example of the salted egg yolk samples used in the test is shown in Figure 12.

For the generalization test dataset, the mAP value of the proposed model for detecting salted egg yolks with impurities reached 0.877. This result demonstrates that the model effectively distinguishes between different quantities of salted egg yolks with impurities in various scenarios, indicating strong generalization ability.

#### 3.1.4. Ablation Experiment

Ablation experiments were conducted to evaluate the effects of varying the number of CA modules on the performance of the YOLOv7-SEY model for detecting salted egg yolks with impurities. Additionally, various attention mechanisms, including SE, CBAM, ECA, and SimAM, were compared with the proposed CA mechanism. The Focal-EIoU loss function was also compared with alternative loss functions, Wise-IoU and Alpha-IoU. The results of these experiments are summarized in Table 3.

As indicated by the data in Table 3, the detection accuracy of the proposed method improves with an increasing number of CA modules. Specifically, incorporating three CA modules with the Focal-EIoU loss function resulted in a higher mAP value compared to using two or one CA module, with increases of 0.004 and 0.021, respectively. However, when only one CA module was used, the mAP value was lower than that of the original YOLOv7, indicating that densely embedding CA modules positively impacts detection accuracy. Furthermore, among the various attention mechanisms evaluated, CA achieved the highest mAP value, surpassing SE, CBAM, ECA, and SimAM. This suggests that CA is more suitable for the salted egg yolk detection task in this study. Moreover, replacing the Focal-EIoU loss function with Wise-IoU and Alpha-IoU led to significant decreases in mAP, by 0.106 and 0.037, respectively. This underscores the advantage of using the Focal-EIoU method for optimizing detection and localization performance in the YOLOv7 model for salted egg yolks with impurities.

#### 3.1.5. Comparison and Analysis

**Figure 13:**
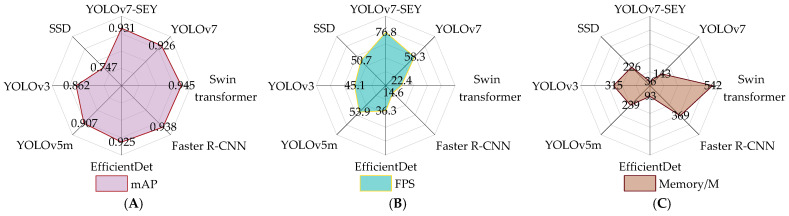
Comparison test results. (**A**) Comparison results of mAP values. (**B**) Comparison results of FPS values. (**C**) Comparison results of optimal model memory.

With the ongoing advancements in artificial intelligence technology and increasing computational power, the landscape of deep learning-based object detection algorithms has broadened significantly. However, variations in experimental conditions often lead to substantial differences in detection performance among these algorithms [31,32]. To evaluate the performance advantages and limitations of the proposed YOLOv7-SEY method for detecting salted egg yolks with impurities, a series of comparative experiments were conducted. These experiments included both two-stage and single-stage algorithms, such as Swin Transformer [33], Faster R-CNN [34], EfficientDet [35], YOLOv5m [36], YOLOv3 [37], and SSD [38]. The comparison focused on accuracy, assessed through mean average precision (mAP), and real-time performance, measured using FPS. To ensure consistency, training hyperparameters and compilation settings were kept identical across all models. The results of these comparisons are illustrated in Figure 13.

The mAP comparison revealed that the Swin Transformer achieved the highest accuracy, followed by Faster R-CNN and then YOLOv7-SEY. Specifically, YOLOv7-SEY demonstrated decreases of 0.014 and 0.007 in mAP compared to the Swin Transformer and Faster R-CNN, respectively. This discrepancy can be attributed to the Swin Transformer’s window-based multi-head self-attention mechanism, which enhances sensitivity to fine eggshell features, along with its effective shifted window multi-head self-attention and multi-layer perception strategies. By contrast, Faster R-CNN employs a region proposal network to generate candidate boxes, which are then classified and regressed. This approach enhances feature recognition and localization compared to YOLOv7-SEY, which predicts object positions and categories directly from feature maps. Additionally, YOLOv7-SEY outperforms EfficientDet, YOLOv5, YOLOv3, and SSD by 0.006, 0.024, 0.069, and 0.184 in mAP, respectively. SSD recorded the lowest mAP value of 0.074, indicating its limited effectiveness in detecting salted egg yolks with impurities in this context. For practical applications, such as in salted egg yolk processing lines, models must balance precision and real-time performance. Although Swin Transformer and Faster R-CNN achieve higher accuracy, YOLOv7-SEY demonstrates superior real-time performance, with FPS values exceeding those of Swin Transformer and Faster R-CNN by 54.4 and 62.2, respectively. This suggests that YOLOv7-SEY achieves a commendable balance between accuracy and real-time performance.

Portability, which is closely related to model size, facilitates deployment on mobile devices. To assess portability and the impact of integrating MobileOne into YOLOv7′s architecture, this study compared memory usage. Figure 13C shows that the YOLOv7-SEY model requires 36 MB of memory, representing reductions of 93.36%, 90.24%, 61.29%, 84.94%, 88.57%, and 84.07% compared to Swin Transformer, Faster R-CNN, EfficientDet, YOLOv5m, YOLOv3, and SSD, respectively. This model is 74.83% smaller than the original YOLOv7, highlighting its superior portability. The integration of MobileOne substantially reduces model parameter size and enhances portability. Overall, considering accuracy, real-time performance, and portability, the YOLOv7-SEY method provides an optimal comprehensive detection capability for salted egg yolks with impurities.

### 3.2. Tracking Results of Salted Egg Yolk Objects with Impurities Based on DeepSORT

#### 3.2.1. Tracking Results of Salted Egg Yolk with Impurities

**Figure 14:**
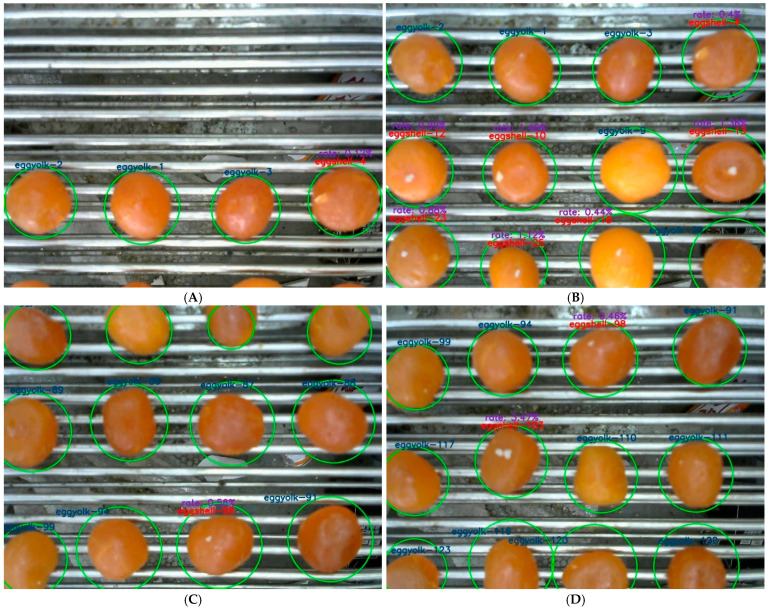

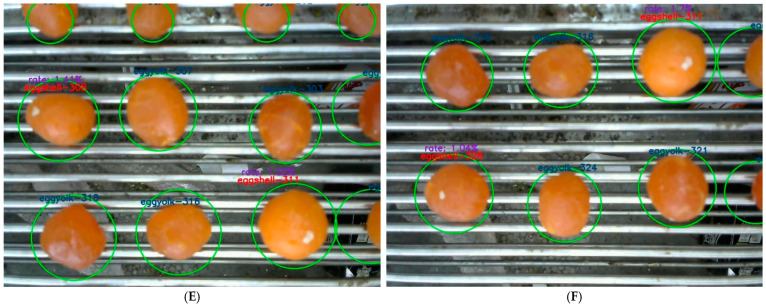
Tracking results of salted egg yolks with impurities based on YOLOv7-SEY combined with DeepSORT. (**A**) Frame 2. (**B**) Frame 24. (**C**) Frame 191. (**D**) Frame 247. (**E**) Frame 447. (**F**) Frame 495.

The proposed method, integrating YOLOv7-SEY for detection with DeepSORT for tracking, was evaluated using datasets of salted egg yolks. Three random segments from online production videos containing these yolks were selected to create a tracking dataset and assess the method’s effectiveness. Tracking results for frames 2, 24, 191, and 247 are illustrated in Figure 14.

During the target tracking phase, rectangular bounding boxes were adjusted to circular shapes to better conform to the contours of the egg yolks, and label string lengths were shortened. As shown in Figure 14, the proposed method effectively differentiated between normal salted egg yolks and those with impurities in the initial stages of Video 1, accurately assigning IDs to the salted egg yolks with impurities. As the production line continued and the number of egg yolk targets increased toward the end of the video, the method maintained precise tracking of the salted egg yolks with impurities, with no ID mismatches. Similarly, in Videos 2 and 3, which had significantly more frames than Video 1, the method continued to accurately track salted egg yolks with impurities targets, correctly maintaining IDs. These results demonstrate the effectiveness of the proposed method in tracking salted egg yolks with impurities, showcasing its ability to consistently and accurately track across varying frame counts.

#### 3.2.2. Comparison and Analysis

**Figure 15:**
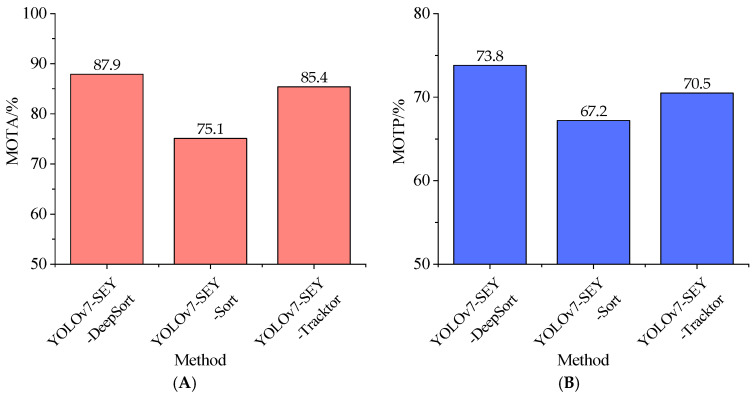
Comparison results of each tracking method for salted egg yolks with impurities. (**A**) Comparison results of MOTA for each tracking method of salted egg yolks with impurities. (**B**) Comparison results of MOTP for each tracking method of salted egg yolks with impurities.

The performance of the YOLOv7-SEY and DeepSORT-based tracking methods was compared with alternatives, including Sort and Tracktor [39]. The evaluation focused on the MOTA and MOTP metrics. During the comparative experiments, frame rates and video durations were kept consistent. The results comparing YOLOv7-SEY-DeepSORT, YOLOv7-SEY-SORT, and YOLOv7-SEY-Tracktor on these metrics are presented in Figure 15.

As illustrated in Figure 15, YOLOv7-SEY-DeepSORT outperforms YOLOv7-SEY-Tracktor, while YOLOv7-SEY-SORT demonstrates the lowest performance. Specifically, YOLOv7-SEY-DeepSORT exhibits improvements of 17.0% and 9.8% in MOTA and MOTP, respectively, compared to YOLOv7-SEY-SORT, and 2.9% and 4.7% compared to YOLOv7-SEY-Tracktor. These improvements are attributed to DeepSORT’s use of deep learning techniques, which combine object detection and feature extraction through convolutional neural networks (CNN), leading to more reliable associations of salted egg yolk data. This reduces erroneous associations and target loss, thereby enhancing tracking stability and distinguishing salted egg yolks with impurities and normal salted egg yolks based solely on appearance. In contrast, SORT primarily relies on simple associations using Kalman filters and appearance features, demonstrating less resistance to interference and reduced accuracy. Tracktor, which integrates appearance and motion information for target association, provides more accurate tracking than SORT but exhibits weaker feature extraction capabilities compared to DeepSORT. Overall, these results indicate that the YOLOv7-SEY combined with DeepSORT delivers precise detection and tracking, outperforming other main-stream tracking methods in this experimental setup.

## 4. Discussion

Salted egg yolks are critical raw materials in various processed snack foods and are experiencing increasing demand. During the shell removal process, small eggshell fragments may become embedded in the surface of salted egg yolks, leading to the presence of impurities. This contamination poses a significant food safety hazard. Timely detection and identification of salted egg yolks with impurities are essential for ensuring food safety, enhancing production automation, and improving industry competitiveness. This paper addresses these issues by proposing an online automatic detection method for salted egg yolks with impurities based on computer vision technology. Additionally, mainstream object tracking algorithms are employed to track these salted egg yolks with impurities. The discussion centers on three main points derived from the experimental process and results.

### 4.1. Effects of Introducing Attention Mechanisms and Optimizing Loss Functions on Model Performance

Numerous studies have demonstrated that targeted improvements in neural network structures, tailored to specific experimental object characteristics and environmental parameters, can enhance model performance. This study addressed the unique characteristics of salted egg yolks with impurities by proposing a detection method based on YOLOv7-SEY. This method incorporates a CA mechanism in the feature fusion section of the YOLOv7 network and replaces the Focal-EIoU loss function. Experimental results indicated that these optimization strategies positively impacted the accuracy of detecting salted egg yolks with impurities, as evidenced by an increased mAP. Comparative experiments further validated the precision benefits of these approaches. Hence, selectively integrating attention mechanisms, substituting loss functions, and deepening networks can improve model performance and detection effectiveness, especially in tasks requiring the identification of significant target features or the enhancement of distinguishing characteristics.

### 4.2. Effects of Lightweight Neural Networks on Model Real-Time Performance

Food processing production lines demand high efficiency, necessitating real-time target detection. To meet this requirement, the original feature extraction network was replaced with the lightweight neural network MobileOne to enhance the real-time performance of the detection model for salted egg yolks with impurities. Experimental results confirm the effectiveness of this approach. However, lightweight neural networks like MobileOne generally exhibit reduced performance in feature extraction and inference compared to deep convolutional neural networks, potentially leading to decreased detection accuracy. In scenarios requiring a balance between accuracy and real-time performance, alternative feature computation methods may be necessary to ensure accurate detection. By incorporating MobileOne alongside attention mechanisms and optimized loss functions, this study achieved a balance between real-time performance and accuracy. These findings provide insights for optimizing both real-time performance and accuracy in other object detection tasks utilizing lightweight neural networks.

### 4.3. Effects of Feature Extraction Effectiveness on Target Tracking Accuracy

In the experimental environment of this study, the effectiveness of tracking salted egg yolks with impurities was compared among Sort, Tracktor, and DeepSORT, in conjunction with YOLOv7-SEY. DeepSORT demonstrated the highest accuracy, attributed to its use of CNN for target identification, tracking, and association, which enhances tracking stability. In contrast, Sort and Tracktor rely on appearance features and motion information but exhibit lower complexity and robustness in feature representation compared to CNN. This results in less effective correlation of salted egg yolks with impurities across frames, highlighting the positive correlation between effective feature extraction and tracking accuracy.

### 4.4. Limitations and Future Direction

Experiments have demonstrated that the salted egg yolks with impurities detection method based on YOLOv7-SEY combined with DeepSORT effectively achieves real-time and stationary detection of salted egg yolks with impurities in production line scenarios. This method also facilitates accurate online tracking with notable precision and real-time performance, surpassing other mainstream approaches. However, the following several limitations remain:

(1) The current process for annotating salted egg yolk targets is labor-intensive and repetitive. Future research should focus on unsupervised learning techniques to eliminate manual annotation, thereby reducing data processing costs and improving detection outcomes.
(2) While the method effectively detects both normal salted egg yolks and those with impurities, it does not provide precise counting of the salted egg yolks. Future research should extend this capability to enable accurate online counting of salted egg yolks.
(3) A pneumatic suction collection system will be designed to automatically clean and collect defective salted egg yolks. This system enhanced the automation level of the production line and improved food safety.

## 5. Conclusions

This study introduced a novel detection method for salted egg yolks with impurities, leveraging YOLOv7-SEY, with YOLOv7 serving as the base network and MobileOne incorporated as the backbone. The proposed method integrated the CA mechanism within the feature fusion process and utilized the Focal-EIoU loss function. Experimental evaluations demonstrated that the method achieved precise detection of salted egg yolks with impurities in production line scenarios, attaining a mAP of 0.931. This result reflects a 0.53% improvement over the baseline YOLOv7 model. The proposed approach effectively balanced high detection accuracy with real-time performance, surpassing other contemporary object detection methods. Additionally, the study employed DeepSORT to develop a tracking framework for salted egg yolks with impurities. This framework yielded MOTA and MOTP scores of 87.9% and 73.8%, respectively, highlighting its efficacy in accurate online tracking of salted egg yolks with impurities. The tracking performance of the YOLOv7-SEY-DeepSORT method was superior to that of Sort and Tracktor, demonstrating its enhanced capability for precise tracking in complex scenarios. Overall, the proposed method contributes significantly to the automation of salted egg yolk processing by improving detection accuracy and real-time performance. It also reduces food safety risks and supports the advancement of intelligent processing applications within the industry.

## Figures and Tables

**Table 1:** The distribution of the dataset of salted egg yolks with impurities.

Labels	Train	Test
Normal salted egg yolks	7984	932
Salted egg yolk with impurities	1431	266

**Table 2:** Hyperparameter settings for training the salted egg yolks with impurities detection model based on YOLOv7-SEY.

Optimizer	SGD
Momentum Factor	0.9
Learning Rate	0.001
Batchsize	16
Epoch	200
Weight_decay	0.0001

**Table 3:** Results of the ablation experiment and comparative test.

YOLOv7-SEY	CA	SE	CBAM	ECA	SimAM	Wise-IoU	Alpha-IoU	Focal-EIoU	mAP
✓	3							✓	**0.931**
✓	2							✓	0.927
✓	1							✓	0.910
✓		✓						✓	0.923
✓			✓					✓	0.915
✓				✓				✓	0.926
✓					✓			✓	0.928
✓	3					✓			0.825
✓	3						✓		0.894

## Data Availability

The original contributions presented in the study are included in the article, further inquiries can be directed to the corresponding author.

## References

[1] Zhao H., Yu H. (2024). Review of China’s poultry egg market in 2023 and outlook of price trend in 2024. Guide Chin. Poult..

[2] Zheng D., Huo J., Li S., Liu X., Lei Y. (2023). Research status, problems and trends of poultry egg processing in China. Guide Chin. Poult..

[3] Li X., Chen S., Yao Y., Wu N., Xu M., Zhao Y., Tu Y. (2022). The quality characteristics formation and control of salted eggs: A review. Foods.

[4] Yao X., Xu J., Adhikari B., Lv W., Chen H. (2022). Mooncake production waste: Nutritional value and comprehensive utilization of salted duck egg white. J. Food Process. Preserv..

[5] Chen H., Chang Y., Chen Y., Liu C., Zhu Z., Liu S., Wang Q. (2024). Nondestructive testing of runny salted egg yolk based on improved ConvNeXt-T. J. Food Sci..

[6] Xu F., Huang X., Tian X., Yu S., Zhang X., Zareef M. (2024). Application of hyperspectral imaging and colorimetric sensor array coupled with multivariate analysis for quality detection during salted duck eggs processing. J. Food Process Eng..

[7] Chen Y., Chen Z., Yan Q., Liu Y., Wang Q. (2024). Non-destructive detection of egg white and yolk morphology transformation and salt content of salted duck eggs in salting by hyperspectral imaging. Int. J. Biol. Macromol..

[8] Tian W., Wang Q., Xu B., Chen Y., Xiao S., Fan W., Lin W., Liu S. (2023). Non-Destructive Detection of Physical and Chemical Indicators of Salted Duck Eggs during Salting Using Near-Infrared Spectroscopy. Food Sci..

[9] Long M., Song Y., Du Q., Zhou D., Cai H., Zhan G. (2015). Effect of pickling temperature and concentration of salt solution on li-pid of duck egg yolk. Trans. Chin. Soc. Agric. Eng..

[10] Li S., Wang S., Wang Y., Liu S. (2019). Design and experiment on temperature control system for quick pickling salty duck eggs. J. Hebei Agric. Univ..

[11] Li Q., Shao Z., Zhou W., Su Q., Wang Q. (2023). MobileOne-YOLO: Improving the YOLOv7 network for the detection of unfertilized duck eggs and early duck embryo development—A novel approach. Comput. Electron. Agric..

[12] Dong J., Dong X., Li Y., Peng Y., Chao K., Gao C., Tang X. (2019). Identification of unfertilized duck eggs before hatching using visible/near infrared transmittance spectroscopy. Comput. Electron. Agric..

[13] Bao G., Jia M., Xun Y., Cai S., Yang Q. (2019). Cracked egg recognition based on machine vision. Comput. Electron. Agric..

[14] Turkoglu M. (2021). Defective egg detection based on deep features and Bidirectional Long-Short-Term-Memory. Comput. Electron. Agric..

[15] Tang Y., Hong Q., Wang Q., Zhu Z. (2018). Sex identification of chicken eggs based on blood line texture features and GA-BP neural network. J. Huazhong Agric. Univ..

[16] Zhu Z., Tang Y., Hong Q., Huang P., Wang Q., Ma M. (2018). Female and male identification of early chicken embryo based on blood line features of hatching egg image and deep belief networks. Trans. Chin. Soc. Agric. Eng..

[17] Liu Y., Xiao D., Zhou J., Zhao S. (2023). AFF-YOLOX: An improved lightweight YOLOX network to detect early hatching information of duck eggs. Comput. Electron. Agric..

[18] Dong J., Lu B., He K., Li B., Zhao B., Tang X. (2021). Assessment of hatching properties for identifying multiple duck eggs on the hatching tray using machine vision technique. Comput. Electron. Agric..

[19] Wang C., Bochkovskiy A., Hong M. YOLOv7: Trainable bag-of-freebies sets new state-of-the-art for real-time object detectors. Proceedings of the Computer Vision and Pattern Recognition (CVPR).

[20] M Badgujar C., Poulose A., Gan H. (2024). Agricultural object detection with You Only Look Once (YOLO) Algorithm: A bibliometric and systematic literature review. Comput. Electron. Agric..

[21] Ariza-Sentís M., Vélez S., Martínez-Peña R., Baja H., Valente J. (2024). Object detection and tracking in precision farming: A sys-tematic review. Comput. Electron. Agric..

[22] Anasosalu Vasu P., Gabriel J., Zhu J., Tuzel O., Ranjan A. MobileOne: An Improved One millisecond Mobile Backbone. Proceedings of the Computer Vision and Pattern Recognition (CVPR).

[23] Hou Q., Zhou D., Feng J. Coordinate attention for efficient mobile network design. Proceedings of the Computer Vi-sion and Pattern Recognition (CVPR).

[24] Zhang Y., Ren W., Zhang Z., Jia Z., Wang L., Tan T. Focal and efficient IOU loss for accurate bounding box regression. Proceedings of the Computer Vision and Pattern Recognition (CVPR).

[25] Qi X., Zhi M. (2024). Review of attention mechanisms in image processing. J. Front. Comput. Sci. Technol..

[26] Li J., Du J., Zhu Y., Guo Y. (2023). Survey of transformer-based object detection algorithms. Comput. Eng. Appl..

[27] Wojke N., Bewley A., Paulus D. Simple online and realtime tracking with a deep association metric. Proceedings of the Computer Vision and Pattern Recognition (CVPR).

[28] Bewley A., Ge Z., Ott L., Ramos F., Upcroft B. Simple online and realtime tracking. Proceedings of the Computer Vision and Pattern Recognition (CVPR).

[29] Zhang Z., Chai X., Si G., Zhang X. (2024). Quantifying variability in zebrafish larvae locomotor behavior across experimental conditions: A learning-based tracker. Fishes.

[30] Kumar S., Singh S.K., Varshney S., Singh S., Kumar P., Kim B.-G., Ra I.-H. (2023). Fusion of deep sort and Yolov5 for effective vehicle detection and tracking scheme in real-time traffic management sustainable system. Sustainability.

[31] Zhao S., Bai Z., Meng L., Han G., Duan E. (2023). Pose estimation and behavior classification of jinling white duck based on improved HRNet. Animals.

[32] Fu X., Zhao S., Wang C., Tang X., Tao D., Li G., Jiao L., Dong D. (2024). Green Fruit Detection with a Small Dataset under a Similar Color Background Based on the Improved YOLOv5-AT. Foods.

[33] Liu Z., Lin Y., Cao Y., Hu H., Wei Y., Zhang Z., Lin S., Guo B. Swin transformer: Hierarchical vision transformer using shifted windows. Proceedings of the International Conference on Computer Vision (ICCV).

[34] Ren S., He K., Girshick R., Sun J. Faster R-CNN: Towards real-Time object detection with region proposal Networks. Proceedings of the Neural Information Processing Systems (NIPS).

[35] Tan M., Pang R.V., Le Q. EfficientDet: Scalable and efficient object detection. Proceedings of the Computer Vision and Pattern Recognition (CVPR).

[36] Jocher G. Ultralytics/Yolov5. https://github.com/ultralytics/yolov5.

[37] Redmon J., Farhadi A. (2018). YOLOv3: An incremental improvement. IEEE Trans. Pattern Anal..

[38] Liu W., Anguelov D., Erhan D., Szegedy C., Reed S., Fu C., Berg A.C. SSD: Single shot multiBox detector. Proceedings of the Computer Vision and Pattern Recognition (CVPR).

[39] Bergmann P., Meinhardt T., Leal-Taixe L. Tracking without bells and whistles. Proceedings of the IEEE Interna-tional Conference on Computer Vision (ICCV).

